# Infection with *Hymenolepis diminuta* Blocks Colitis and Hastens Recovery While Colitis Has Minimal Impact on Expulsion of the Cestode from the Mouse Host

**DOI:** 10.3390/pathogens10080994

**Published:** 2021-08-06

**Authors:** Shuhua Li, Sruthi Rajeev, Arthur Wang, Derek M. McKay

**Affiliations:** Gastrointestinal Research Group, Inﬂammation Research Network and Host-Parasite Interaction Group, Department of Physiology and Pharmacology, Calvin, Phoebe & Joan Snyder Institute for Chronic Diseases, Cumming School of Medicine, University of Calgary, Calgary, AB T2N 4N1, Canada; shuhua.li@ucalgary.ca (S.L.); sruthi.rajeev1@ucalgary.ca (S.R.); fhawang@ucalgary.ca (A.W.)

**Keywords:** colitis, helminth therapy, cestode immunomodulation, DNBS colitis therapeutic, Th2 cytokines

## Abstract

Two experimental paradigms were adopted to explore host–helminth interactions involved in the regulation of colitis and to understand if colitis affects the outcome of helminth infection. First, male BALB/c mice infected with *H. diminuta* were challenged 4 days later with dinitrobenzene sulphonic acid (DNBS) and necropsied 3 days later. Second, mice were infected with *H. diminuta* 3 days after DNBS treatment and necropsied 11 or 14 days post-DNBS. Mice were assessed for colitic disease severity and infectivity with *H. diminuta* upon necropsy. Supporting the concept of helminth therapy, mice are protected from DNBS–colitis when infected with *H. diminuta* only 4 days previously, along with parallel increases in splenic production of Th2 cytokines. In the treatment regimen, *H. diminuta* infection produced a subtle, statistically significant, enhanced recovery from DNBS. Mice regained body weight quicker, had normalized colon lengths, and showed no overt signs of disease, in comparison to the DNBS-only mice, some of which displayed signs of mild disease at 14 days post-DNBS. Unexpectedly, colitis did not affect the hosts’ anti-worm response. The impact of inflammatory disease on helminth infection is deserving of study in a variety of models as auto-inflammatory diseases emerge in world regions where parasitic helminths are endemic.

## 1. Introduction

The increased prevalence of auto-inflammatory conditions, such as diabetes, arthritis, multiple sclerosis, and inflammatory bowel disease (IBD), coupled with a lack of cures for these conditions underscores the need for innovative approaches to manage idiopathic disease [1]. Epidemiological studies demonstrating an inverse relationship between the global distribution of endemic parasitic helminths and regions of high incidence of auto-inflammatory disease, suggest the possibility of novel helminth-based therapy for IBD and other allergic autoimmune disorders [2]. Indeed, use of animal models of disease, especially colitis, has repeatedly shown that infection with a variety of parasitic helminths can reduce the severity of inflammatory disease [3,4,5,6,7].

The rat tapeworm, *Hymenolepis diminuta*, is an intriguing candidate as a ‘therapeutic helminth’. Infection is by ingestion and the worm does not migrate through the host; rather, it seeks to establish in the small intestine. Bearing no teeth or hooks, it does no obvious abrasive damage to the host. It is not auto-infective and its life-cycle requires an invertebrate host, so there is no direct person-to-person spread. Natural infection is rare in humans, typically restricted to malnourished or immunocompromised individuals and can be treated with anthelmintics [8]. Studies with *H. diminuta* in the dextran sodium sulphate (DSS) [9] and di-nitrobenzene sulphonic acid (DNBS) [3] murine models of colitis were among the first to provide proof-of-concept data in support of helminth therapy to reduce the severity of colonic inflammation and concomitant signs of disease. While ongoing studies have confirmed the anti-colitic effect in the DNBS-model and revealed some of the nuances of the helminth–host interaction [10,11,12], two major questions remain unaddressed: both germane to helminth therapy: First, what is the temporal window of opportunity for *H. diminuta* to suppress colitis? Elucidation of the kinetics of infection in the context of inflammation can provide insight into host–parasite interaction and potentially reveal new targets for therapeutic intervention. Second, does intestinal inflammation affect the immune response to the helminth? This is an important, yet largely ignored issue. For instance, as the incidence of IBD increases in under-developed countries [13], will this counter helminth infection or render the population more vulnerable to parasitic helminths?

Using an acute and spontaneously resolving colitis model, we address these two questions. The results herein show that mice challenged with DNBS four days after infection with *H. diminuta* developed less severe colitis. Reciprocally, DNBS–colitis had negligible effects on the outcome of infection; mice still mobilized a Th2 response and expelled a five-worm burden by 11 days post-infection (dpi). These data are consistent with the speculation that the emergence of IBD in under-developed nations, as they adopt a ‘westernized lifestyle’, could be slowed by the presence of parasitic helminths, and that the immune response against these ‘old friends’ [14] may not be deleteriously affected.

## 2. Results

### 2.1. H. diminuta Infection Four Days Prior to DNBS Suppresses Colitis

**Figure 1:**
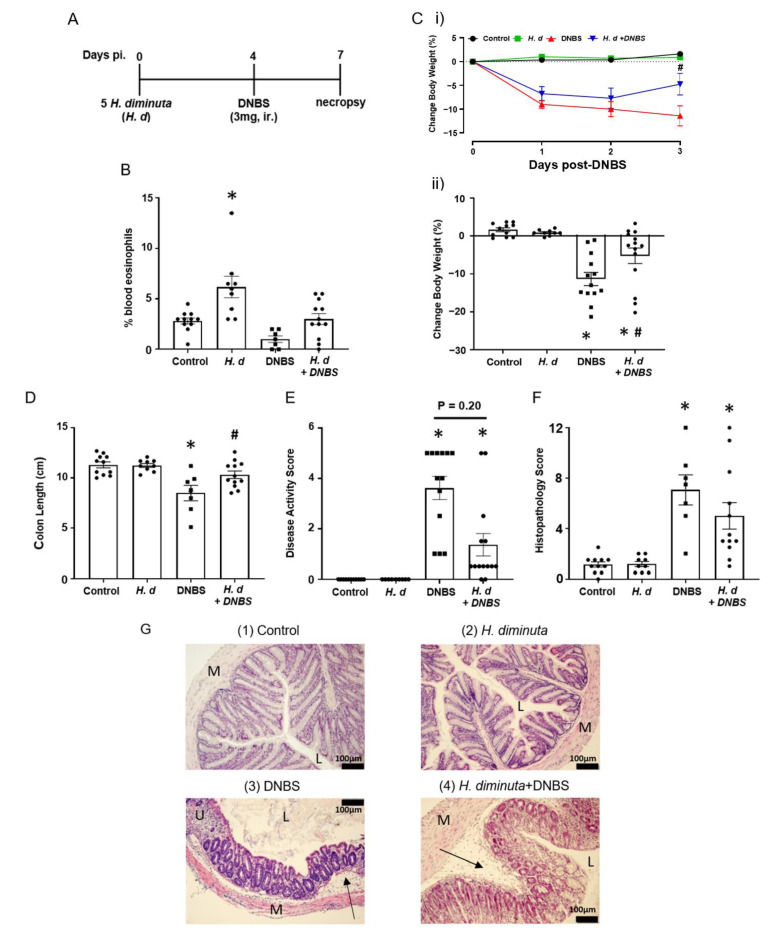
Infection with *H. diminuta* four days prior to DNBS alleviates the severity of colitis. Male BALB/c mice were gavaged with 5 cysticercoids of *H. diminuta*, challenged with DNBS (3 mg, ir.) 4 days later and necropsied at 3 days post-DNBS (**A**), when blood eosinophilia confirmed helminth infection (**B**). Disease was assessed by body weight (**C**). (**i**), daily change post-DNBS; (**ii**), final change, colon length (**D**), disease activity score (**E**), and histopathology scores (**F**) (representative H & E images shown in (**G**)) (data are mean ± SEM; *n* = 7–12 mice pooled from 3 experiments; parametric data analyzed by one-way ANOVA and Tukey’s multiple comparisons test and non-parametric data analyzed by a Kruskal–Wallis test with Dunn’s post-test; * and #, *p* < 0.05 compared to control and DNBS only, respectively; *H. d*, *H. diminuta*; panel G, sections (M: muscle; L: gut lumen; U: ulcer; arrow, inflammatory infiltrate)).

Previous studies on the impact of infection with *H. diminuta* on DNBS-induced colitis have focused on 8-dpi based on the rationale that at this time point anti-worm immune responses have been mobilized (e.g., upregulation of Th2 cytokines) and a bystander effect of this would be modulation of colitis [3,15]. Mice that received *H. diminuta* 4 days prior to DNBS (Figure 1A) displayed less colitis upon necropsy at 72 h post-DNBS (i.e., 7-dpi with *H. diminuta*) (successful worm infection, current or recently expelled, was confirmed by blood eosinophilia (Figure 1B)). Typical of this model, mice challenged with DNBS displayed decreased activity and ruffled fur and disease in 6 of 13 mice (i.e., 46%) was so severe it required humane euthanization, whereas *H. diminuta* + DNBS-treated mice appeared healthier with only 2 of 14 mice (14%) reaching a disease severity pre-determined end-point requiring euthanasia. The drop in body weight and shortening of the colon caused by DNBS were significantly less in *H. diminuta*-infected mice compared to DNBS-only treated mice (Figure 1C,D). The average disease activity score was reduced by ~50% in the *H. diminuta* + DNBS group, although this was not statistically significant compared to the DNBS-only group, possibly due to inclusion of mice that required euthanization in this overall assessment of disease (Figure 1E). Inspection of colon sections revealed loss of crypt architecture, significant inflammatory cellular infiltration, edema, ulceration, and circumferential tissue damage in the colon of mice given DNBS. While 7 of 12 *H. diminuta* + DNBS-treated mice had mild histopathology, 2 mice in the group had major colonic ulceration, resulting in a lack of a statistically significant improvement in histopathology in this group as compared to the DNBS-only group (Figure 1F,G). While we think it unlikely since *H. diminuta* seeks to lodge in the small intestine and DNBS is given intra-rectally (i.e., limited chance of direct contact), the possibility that the stunted *H. diminuta* or enteric changes evoked in response to the infection affects the haptenizing ability of DNBS has not been excluded. 

**Figure 2:**
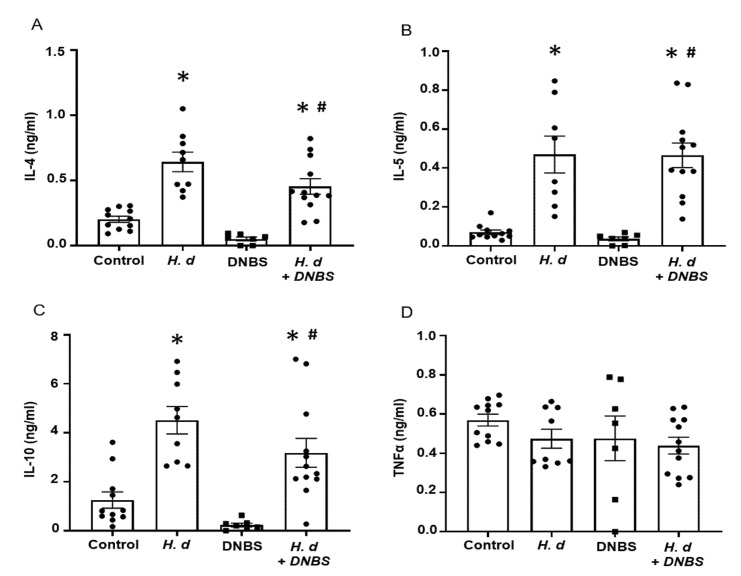
Infection with *H. diminuta* evokes increased splenic production of Th2 cytokines. Male BALB/c mice were gavaged with 5 cysticercoids of *H. diminuta*, challenged with DNBS (3 mg, ir.) 4 days later and on necropsy 3 days later (see Figure 1A), splenocytes were isolated and stimulated with concanavalin-A (2 µg/5 × 10^6^ splenocytes; 48 h) and IL-4 (**A**), IL-5 (**B**), IL-10 (**C**), and TNFα (**D**) measured by ELISA (data are mean ± SEM; *n* = 7–12 mice pooled from 3 experiments; one-way ANOVA and Tukey’s multiple comparisons test; * and #, *p* < 0.05 compared to control and DNBS only, respectively; *H. d*, *H. diminuta*).

Using mitogen-stimulated splenocytes as a marker of systemic immunity revealed increased IL-4, IL-5, and IL-10 production by cells from *H. diminuta*-infected mice ± DNBS (Figure 2A–C). These data confirmed successful infection with the helminth and corroborated earlier studies that implicated IL-10 in the anti-colitic effect [3]. In contrast, splenocytes from DNBS-treated mice had reduced IL-10 output following challenge with concanavalin-A (Figure 2C). Production of the pro-inflammatory cytokine, TNFα, was not different between the groups (Figure 2D). It is important to supplement these data with a consideration of local (i.e., mesenteric lymph node, lamina propria lymphocytes) immunity in this model paradigm as well as an assessment of immunoregulatory factors (e.g., argianse1^+^ regulatory macrophages, Foxp3^+^ regulatory T cells).

### 2.2. DNBS–Colitis Prior to H. diminuta Infection Subtly Affected Worm Expulsion

**Figure 3:**
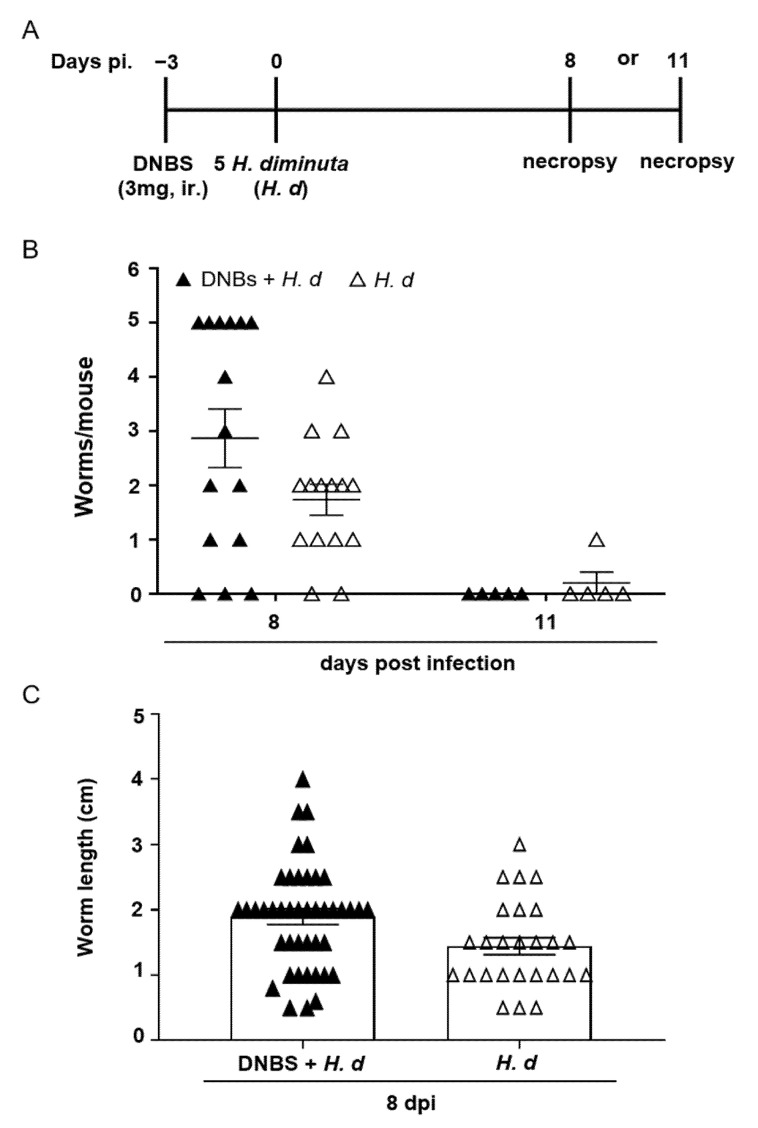
DNBS–colitis subtly affects expulsion of *H. diminuta* from mice. Male BALB/c mice were treated with DNBS (3 mg, ir.) 3 days before infection with 5 cysticercoids of *H. diminuta*, and necropsied 8 or 11 days post-infection (dpi) (**A**). Panel (**B**) shows worm recovery at 8- and 11-dpi and panel (**C**) the length of the worms recovered in intestinal washings at 8-dpi (data are mean ± SEM; *n* = 15 mice at 8-dpi (pooled from 3 experiments) and *n* = 5 mice at 11-dpi from 1 experiment; *H. d, H. diminuta*).

Few studies have examined the effects of colitis on the kinetics of infection or expulsion of a helminth parasite. Thus, mice were treated with DNBS, infected with *H. diminuta* 72 h later and assessed 8- and 11-dpi (Figure 3A). Prior treatment with DNBS had a negligible effect on rejection of *H. diminuta*, with one exception: at 8-dpi, 6 of 15 mice (40%) retained 5 worms (i.e., the full infective burden, although small and stunted (Figure 3B)), and this was not observed in the *H. diminuta*-only group (0/15 mice) (Figure 3B). There was no significant difference in average length of the worms retrieved between the two groups of mice (Figure 3C). By 11-dpi, as is typical of immunocompetent mice [16], the small intestine of DNBS + *H. diminuta* treated mice harbored no worms (Figure 3B).

**Figure 4:**
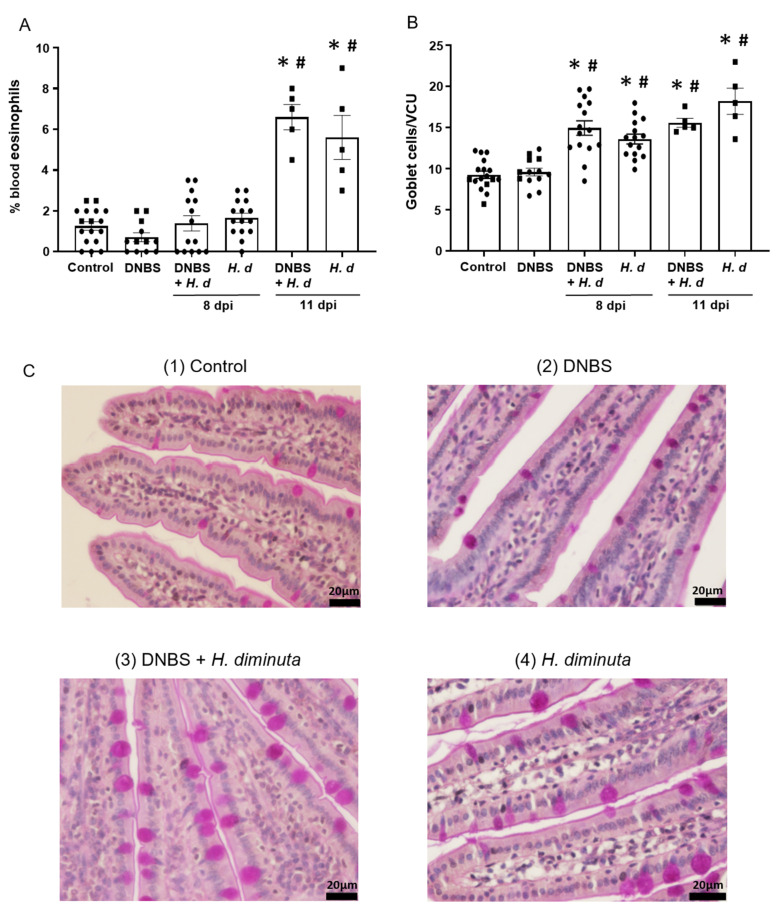
Colitis does not affect the hosts’ effector response to *H. diminuta*. Male BALB/c mice were treated with DNBS (3 mg, ir.) 3 days before infection with 5 cysticercoids of *H. diminuta*, and necropsied 8 or 11 days post-infection (dpi) (see Figure 3A), when blood eosinophils (**A**) and goblet cells in a section of mid-jejunum (**B**) were enumerated (data are mean ± SEM; *n* = 7–15 mice (pooled from 3 experiments) for 8-dpi and *n* = 4–5 mice for 11-dpi; one-way ANOVA and Tukey’s multiple; * and #, *p* < 0.05 compared to control and DNBS only, respectively; VCU, villus crypt unit; *H. d, H. diminuta*). Panel (**C**) presents representative photomicrographs of goblet cell (magenta) staining in jejunal villi.

**Figure 5:**
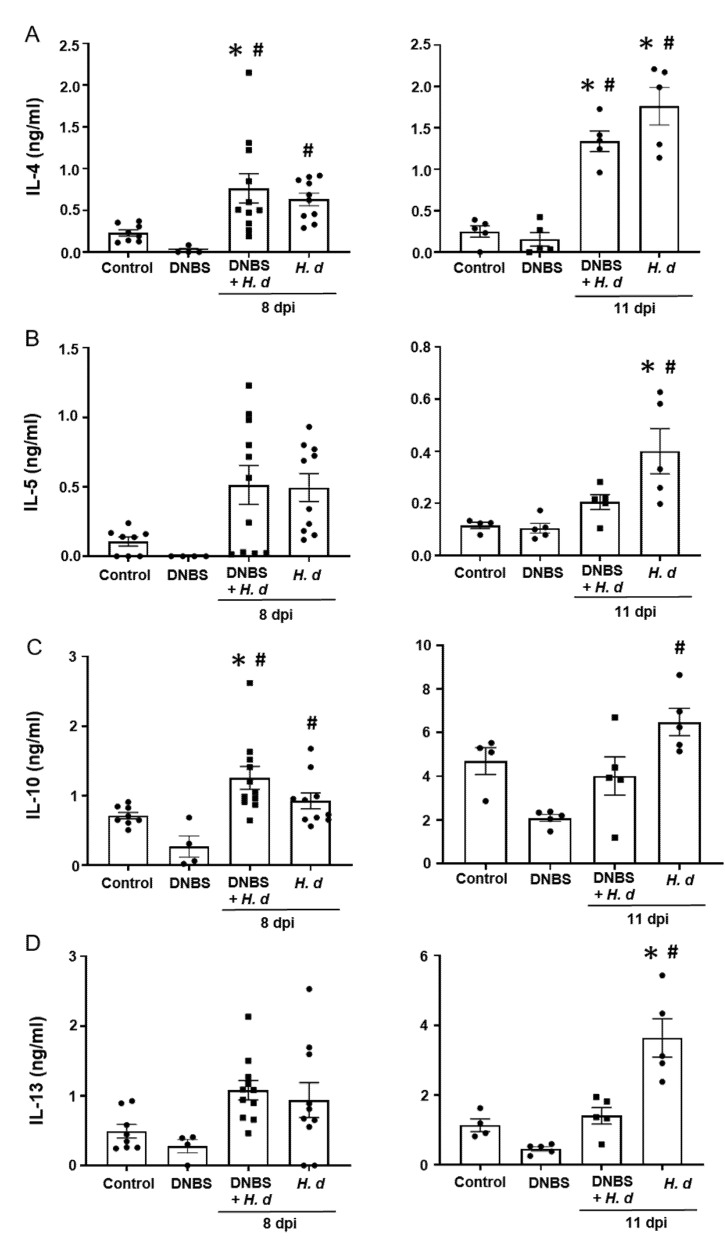
Spleen cell production of TH2 cytokines following infection with *H. diminuta* is not affected by colitis. Male BALB/c mice were treated with DNBS (3 mg, ir.) 3 days before infection with 5 cysticercoids of *H. diminuta*, and necropsied 8 or 11 days post-infection (dpi) (see Figure 3A). Splenocytes were isolated and stimulated with concanavalin-A (2 μg/5 × 10^6^ splenocytes; 48 h) and IL-4 (**A**), IL-5 (**B**), IL-10 (**C**), and IL-13 (**D**) measured by ELISA (data are mean ± SEM; *n* = 4–11 mice (pooled from 2 experiments) for 8-dpi and *n* = 4–5 mice for 11-dpi; one-way ANOVA and Tukey’s multiple comparisons test; * and #, *p* < 0.05 compared to control and DNBS only, respectively; *H. d, H. diminuta*).

From a Th2 immunological perspective, a 72-h pre-treatment with intra-rectal DNBS had minimal impact on the systemic murine response to *H. diminuta*. Blood eosinophils and jejunal goblet cell numbers were increased at 8- and 11-dpi in both *H. diminuta* and *H. diminuta* + DNBS-treated mice (Figure 4A–C). However, variation in cell counts in this experiment meant that control mice and 8-dpi *H. diminuta*-mice were not statistically different in blood eosinophil numbers; a finding that underscores the importance of including time-matched controls in murine experiments. Spleen cells from *H. diminuta*-infected mice (8- and 11-dpi) or DNBS + *H. diminuta* treated mice challenged with Con-A produced increased amounts of IL-4, IL-5, IL-10, and IL-13 compared to splenocytes from control mice or those given DNBS-only (Figure 5).

### 2.3. In the DNBS Model of Colitis Infection with H. diminuta Hastens Recovery

**Figure 6:**
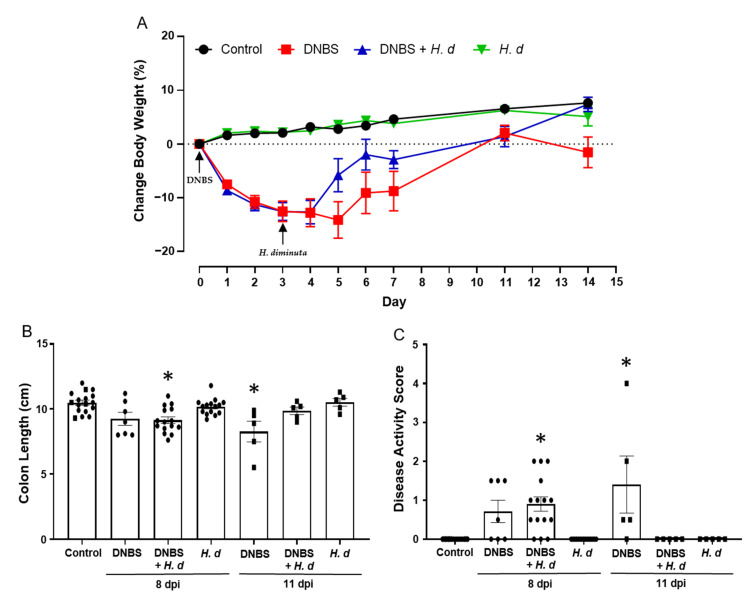
Infection with *H. diminuta* enhances recovery from colitis. Male BALB/c mice were treated with DNBS (3 mg, ir.) 3 days before infection with 5 cysticercoids of *H. diminuta*, and necropsied 8 or 11 days post-infection (dpi) (see Figure 3A). Body weight was recorded daily (**A**), and on necropsy colon length was measured (**B**) and disease activity score calculated (**C**) (data are mean ± SEM; *n* = 7–15 mice (pooled from 3 experiments) for 8-dpi; *n* = 4–5 mice for 11-dpi; parametric data analyzed by one-way ANOVA and Tukey’s multiple comparisons test and non-parametric data analyzed by a Kruskal–Wallis test with Dunn’s post-test; *, *p* < 0.05 compared to control; *H. d*, *H. diminuta*).

The experimental set-up shown in Figure 3A, while designed to assess if DNBS-induced colitis affected *H. diminuta* infectivity, afforded the opportunity to assess if *H. diminuta* in a treatment regimen hastened recovery from DNBS-induced colitis. Mice spontaneous recover from DNBS-induced colitis, as demonstrated by a return to normal behavior and recovery of body weight (pers. observation; Figure 6A). There is natural variability in the murine response to DNBS, and on necropsy at 11 days post-DNBS, mice had returned to their pre-treatment body weight, whereas those treated with DNBS + *H. diminuta* not only gained back the weight, but also continued to thrive and were not different from control naïve mice at 14 days post-DNBS (Figure 6A). Similarly, while mice treated 14 days previously with DNBS displayed mild signs of disease, these were not observed in the DNBS + *H. diminuta* group of mice (Figure 6A–C). Mice gavaged with *H. diminuta*, 3 days after DNBS, were indistinguishable from controls when disease was assessed 14 days after DNBS as gauged by recovery of body weight, colon length, and a zero-disease activity score (Figure 6).

## 3. Discussion

The concept of helminth-therapy to treat idiopathic auto-inflammatory disease is intriguing because it seeks to harness eons of host–parasite co-evolution—that is, the hosts’ natural immune response to infection with a parasitic helminth has the bystander effect of affecting the course of concomitant disease [2,17,18]. While seemingly counterintuitive, Desowitz (1980) elegantly presented the concept of the “Harmonious Parasite” [19] and numerous studies with helminth–rodent model systems have shown that deliberate infection with parasitic helminths can reduce inflammation [20,21,22]. Furthermore, a variety of helminth-derived molecules have immunoregulatory, immunosuppressive, and anti-inflammatory effects [23,24,25].

The consensus on the mechanism of helminth therapy is essentially two-fold: Infection with parasitic helminths drives Th2-dominated immunity and this reduces disease caused by Th1 immunopathology, or mobilization of immunoregulatory cells/factors inhibits inflammation (e.g., Foxp3^+^ regulatory T cells (Tregs), alternatively activated macrophages (AAMs), IL-10, transforming growth factor β (TGFβ)) [21,26]. We showed that infection of the non-permissive murine host with *H. diminuta* 8 days prior to intra-rectal DNBS resulted in significantly less colitis, and that systemic administration of neutralizing IL-10 antibodies negated the anti-colitic effect of infection with this helminth [3]. *H. diminuta* is particularly potent in this system, where gavage with a single cysticercoid led to substantial inhibition of colitis [27]. Typically, much larger worm burdens (e.g., *Schistosoma mansoni*, *Heligmosomoides polygyrus*) have been used to alleviate colitis or other inflammatory conditions [5,28].

Three-week-old mice infected with *H. diminuta* were also protected from DNBS-induced colitis; however, the kinetics of this response differed from adult mice: young mice displayed less colitis when infected 10 days, but not 8 days, prior to treatment with DNBS [10]. This delay in the anti-colitic effect could be due to immaturity of the immune system in the young mouse, and it draws attention to the importance of the kinetics of the infection-colitis regimen. Having rationalized that the anti-colitic effect of infection with *H. diminuta* could be prominent at the peak time of worm expulsion from the mouse host, we have previously focused on 8-dpi when systemic and local immune Th2 responses are apparent [3,29]. In this paradigm, significant and consistent suppression of DNBS-induced colitis is observed; moreover, we noted that protection against this form of colitis occurred in mice infected 14 days prior to DNBS treatment, although to a lesser extent [3]. The current study shows that DNBS–colitis is less severe in mice infected with *H. diminuta* 4 days previously; however, the effect is less pronounced than that observed in mice infected 8 days previously [3]. While we did not pursue the mechanism of the protection against colitis, the similarities in the splenic response at 4- and 8-dpi imply a common mode of action involving IL-4 and IL-10 signaling in the blockade of colitis, as we previously determined [30]. Collectively, these findings show that mice challenged with DNBS between 4–14 dpi with *H. diminuta* develop less severe disease.

While underscoring the capacity of helminth therapy to ameliorate colitis, the current study, like the majority of studies in this area, used a prophylactic protocol. Small clinical trials in which individuals with IBD were treated with ova of the pig whipworm, *Trichuris suis*, have reported improvement in objective and subjective measures of colitis and disease symptoms [31]. Despite the spontaneous resolution of colitis in the DNBS model, we showed that *H. diminuta* given two days after DNBS hastened recovery from disease over a seven-day period [3]. Corroborating and extending these data we find that while some mice displayed signs of mild disease 14 days post-DNBS, this was not observed in mice infected with *H. diminuta* 3 days post-DNBS (i.e., 11 days prior to necropsy). Although these data support the concept of a therapeutic helminth, a recent ~250 IBD-patient trial with *T. suis* ova concluded that while safe the treatment was ineffective [32], potentially sounding the death knell for helminth therapy. However, Crohn’s disease is a heterogeneous condition and with broad inclusion criteria for enrolling patients in the trial it is perhaps naïve to think that a single species of parasitic helminth could benefit all patients: There was also a high placebo effect in the trial [32]. Similarly, helminth therapy has been tested in multiple sclerosis and found to be safe; however, any beneficial “effect was modest” with considerable variation between subjects [33,34], with an inter-individual variability also seen in *T. suis*-specific T and B cell responses [35]. In addition, it is increasingly apparent that infection with enteric parasitic helminths affects the composition and activity of the gut microbiota [5,16,36], the production of short chain fatty acids by which has been implicated as a mediator of helminth-initiated anti-inflammatory effects [28,37]. Furthermore, dysbiosis is a common feature of IBD [38] and how this would affect helminth-therapy is unknown. Thus, we suggest it is premature to abandon the potential of helminth therapy and that in-depth analyses of helminth infection in murine models of disease will aid in unraveling the complexity of immunoregulation, with the potential to identify targets for therapeutic intervention in auto-inflammatory disease.

Using *H. diminuta* in a treatment regimen afforded the opportunity to ask if enteric inflammation affected the course of infection/rejection of the worm. The expectation that DNBS-induced colitis that can be Th1 immune skewed [21] would render mice more susceptible to *H. diminuta*, allowing a longer duration of infection and/or increased biomass was incorrect. While the fact that a number of DNBS + *H. diminuta*-treated mice bore five worms at 8-dpi is noteworthy, and something we have never seen in immunocompetent mice, there was a negligible impact on the Th2 response to infection, as gauged by blood eosinophilia, goblet cell hyperplasia, and spleen cell production of IL-4, -5, -10, and -13, and worms were not detected at 11-dpi. While many studies have sought to dissect the interplay of co-infection, in both helminth–helminth [39] and helminth–microbe [40,41,42] there is a paucity of data on how a pre-existing inflammatory condition would affect the outcome of infection with helminth parasites. This understudied area is more than an intriguing issue of complex immune crosstalk, it is likely to emerge as a clinically relevant topic should the incidence of IBD and other auto-inflammatory diseases increase in global regions of endemic helminth infections. Furthermore, while we found that DNBS–colitis did not affect the hosts’ ability to expel the worm, it is critical that these findings be complemented by other rodent–helminth model systems to support or counter these data, and human epidemiological studies performed to assess relationships between inflammatory disease and subsequent susceptibility to helminth infection.

In summary, in advancing our understanding of host–parasite interactions we found that: (1) Mice challenged with DNBS 4-dpi with *H. diminuta* developed less severe disease; (2) *H. diminuta* hastened the recovery from DNBS–colitis when delivered in a therapeutic regimen; and, (3) colitis did not appreciably affect the ability of the mouse to mount Th2 immune responses and effectively expel the helminth. In total, the findings herein offer additional support for helminth therapy to ameliorate enteric inflammation. However, further studies with this, and other model systems, are needed to fully delineate the anti-inflammatory mechanism(s) evoked following infection with helminth parasites, responses that may be mediated or fine-tuned by participation of the gut microbiota.

## 4. Materials and Methods

### 4.1. Mice, Helminth and DNBS-Induced Colitis

Male Balb/c mice (7–9 weeks of age; Charles River Animal Supplies, Saint-Constant, QC, Canada) were housed in filter-topped cages with free access to food and water on a 12:12 h light: dark cycle. *Hymenolepis diminuta* lifecycle was maintained by cyclic passage through rats (permissive host) and the flour beetle, *Tribolium confusum* (intermediate host). Mice received five infective cysticercoids of *H. diminuta* via oral gavage in 100 µL of 0.9% NaCl [3,43]. 

Colitis was induced in anesthetized mice by intra-rectal (ir.) instillation of 3 mg of di-nitrobenzene sulphonic acid (DNBS; Sigma Chemical Co., St. Louis, MI, USA) in 100 µL of 50% ethanol (EtOH), delivered 3 cm into the colon via a polyethylene catheter [3]. In the first experimental paradigm, mice were infected with *H. diminuta* 4 days prior to DNBS treatment, followed by necropsy 72 h later. In a second paradigm, mice were challenged with DNBS 72 h before *H. diminuta* infection, followed by necropsy at either 8 or 11 days post-infection (dpi). Controls consisted of naïve, *H. diminuta*-infected only, and DNBS-treated only mice. 

### 4.2. H. diminuta Infectivity, Goblet Cells, and Eosinophils

Following humane euthanasia, the entire small intestine was excised from previously infected mice and was flushed with 10 mL PBS at room temperature. This intestinal wash was collected in a Petri dish and extensively examined for the presence of *H. diminuta* (typically stunted and damaged in the mouse). The number of worms was counted and each helminth was measured [44].

A blood drop was smeared onto a coded slide (VWR Micro Slides (frosted selected precleaned)), allowed to air dry, stained with Wright–Giemsa hematology solution, and eosinophils counted in a blinded fashion in a random selection of 200 white blood cells [45].

A ~2-cm segment of mid jejunum was excised, immersion-fixed in 10% neutral buffered formalin for 72 h, then dehydrated and embedded in paraffin wax for transverse sectioning. Five-µm sections were collected onto coded slides and stained by the Schiff’s periodic acid (Sigma Chemical Co., St. Louis, MI, USA) method for goblet cell enumeration. Goblet cells were counted on a per villus-crypt unit (VCU) basis, composed of a crypt opening to the lumen and a round, intact villus tip [43].

### 4.3. DNBS-Induced Colitis

Mice were examined daily following treatment with DNBS for signs of ill health: wet/feces-stained or bloody anus, weight loss, ruffled fur, and hunched, inactive posture. Upon necropsy, the colon was excised, measured, and inspected for signs of inflammation/dysfunction: loose stool, fluid accumulation, bleeding, or ulceration. A disease activity score (0–5 points) was determined based on the following criteria: >10% loss of body weight (0 or 1); wet anus, soft stool, or empty colon (0–1); anal bleeding (0 or 1); macroscopic ulcers present in the colon (0 or 1) [46]. Mice that reached a predetermined end-point (e.g., >20% loss of body weight, rectal prolapse) prior to 72 h post-DNBS (the experimental endpoint) were euthanized and if this occurred 24–72 h post-DNBS, mice were allocated a score of 5 [46]. The excised colon was subsequently divided based on length and the 10–30% segment distant from the anus was formalin-fixed for further histological analysis. 

### 4.4. Colonic Histopathology Assessment

Mid-colonic sections were excised, fixed, paraffin-embedded, cut into 5-µm sections on coded slides and stained with hematoxylin and eosin (H & E). The histopathology score is determined on a 12-point scale and considers degree of loss of architecture, goblet cell depletion, inflammatory cells infiltrate, muscle thickening, edema, and ulceration [3].

### 4.5. Cytokine Production

Spleens were removed and aseptically separated into single-cell suspensions. Five million splenocytes were incubated with concanavalin A (conA, 2 µg/mL) for 48 h at 37 °C, and cytokine levels (IL-4, -5, -10, -13, TNFα) in the supernatants were measured by ELISA (Duo-set kits, R&D Systems) following the manufacturer’s instructions [47].

### 4.6. Statistical Analysis

Data are presented as the mean ± the standard error of the mean (SEM), where *n* is the number of mice. Statistical comparisons for two groups were conducted with a Students’ unpaired *t*-test. Multiple group comparisons were performed via one-way ANOVA followed by Tukey’s test for parametric data or Kruskal–Wallis statistics followed by Dunn’s post-test for non-parametric data, with *p* < 0.05 set as the level of acceptable statistical difference [46].

## Data Availability

Original data are available from authors. There are no database data in this paper.
